# Development and Rapid Sensory Descriptive Characterization of Cereal Bars Made with Brazilian Licuri Nut (*Syagrus coronata*)

**DOI:** 10.3390/foods13030502

**Published:** 2024-02-05

**Authors:** Maximiliano Sommo, Lorena Andrade de Aguiar, António Raposo, Ariana Saraiva, Edite Teixeira-Lemos, Cláudia Chaves, Bernardo Romão

**Affiliations:** 1 Instituto de Educação Superior de Brasilia, IESB University Center, Brasília 70200-730, Brazil; maximiliano.sommo@iesb.edu.br (M.S.); lorena.aguiar@iesb.edu.br (L.A.d.A.); 2 CBIOS (Research Center for Biosciences and Health Technologies), Universidade Lusófona de Humanidades e Tecnologias, Campo Grande 376, 1749-024 Lisboa, Portugal; 3 Department of Animal Pathology and Production, Bromatology and Food Technology, Faculty of Veterinary, Universidad de Las Palmas de Gran Canaria, Trasmontaña s/n, 35413 Arucas, Spain; ariana_23@outlook.pt; 4 CERNAS Research Centre, Polytechnic University of Viseu, 3504-510 Viseu, Portugal; etlemos3@gmail.com; 5 ESSV, Centre for Studies in Education and Innovation (CI&DEI), Polytechnic University of Viseu, 3504-510 Viseu, Portugal; claudiachaves21@gmail.com; 6 Faculty of Health Sciences, Department of Nutrition, University of Brasília, Brasília 70910-900, Brazil

**Keywords:** licuri, cereal bars, acceptance test, CATA

## Abstract

Licuri (*Syagrus coronata*) is an oilseed fruit common in the Brazilian caatinga and cerrado biomes. This fruit has high socioeconomic importance in the regions where it grows, being incorporated into exported animal feed and also into gastronomic preparations. Cereal bars are ready-to-eat highly consumed products with increased demand, commonly made with cereals and oilseeds such as licuri. In this sense, the incorporation of licuri in cereal bars may increase its socioeconomic value and expand its potential use. Thus, the objective of the study was to analyze acceptance and describe the sensory characteristics of cereal bars incorporated with licuri nuts. This study was conducted in four stages: (1) development of samples; (2) chemical composition analysis; (3) sensory analysis; and (4) statistical analysis. Cereal bars with licuri presented proportionally lower carbohydrate and protein content as the incorporation of licuri nut increased. However, the dietary fiber content increased. Further, 122 untrained panelists participated in the analysis. The results showed that samples with all proportions of incorporation of licuri nuts were acceptable. Furthermore, the sensory descriptors related to the presence of licuri were positively associated with product acceptance. In this way, this study demonstrates yet another possibility for use of the fruit, increasing its socioeconomic potential.

## 1. Introduction

The search for foods that offer nutrition and functional benefits is on the rise, as people seek information and seek to improve their eating habits [1]. The trend towards consuming healthier foods has opened space for developing products with functional, ready-to-eat ingredients, such as cereal bars [2].

Cereal bars stand out as unique food products, comprising processed cereal grains that can be enriched with various components, such as whole grains, dehydrated or candied fruits, chestnuts, walnuts, almonds, sugar, confectionery, and other ingredients [3]. The careful combination of these elements plays a fundamental role in ensuring harmonious integration, addressing aspects, such as flavor, texture, and physical characteristics, including the balance of water activity [4,5].

In the early 2000s, the consumption of cereal bars in Brazil began to gain prominence, given that in 2005, cereal bar consumption grew considerably, reaching the mark of 464 million units, with a notable increase of 32% compared to the previous year [6]. This indicated a growing interest in these products in the Brazilian market, with national companies competing for a share of a market valued at RRL 80 million at the time [6]. However, the phenomenon of cereal bars is far from being exclusively Brazilian. 

Looking ahead, the global outlook for cereal bars is equally impressive. The global market is expected to grow, with a compound annual growth rate of 8.5% predicted between 2021 and 2026, surpassing the overall chocolate market by 4% [6]. This increase reflects the growing popularity of cereal bars, representing a global market worth around USD 4 billion, driving the diversification of products, labels, and ingredients to attract increasing consumers [7].

In the rich native flora of Brazil, there are several species that, although not widely known, have great potential in the almond and chestnut market, ingredients commonly used in cereal bars [8]. The *Cerrado* and *Caatinga* are Brazilian biomes with a wide diversity of vegetables and unknown varieties of spontaneous food plants, which may have nutritional, environmental, and commercial relevance [8,9].

Licuri (*Syagrus coronata*) is an oily fruit from the Aracaceae family, native and endemic to Brazilian territory [10]. Resulting from a palm tree, licuri grows in arid conditions in the Brazilian “Caatinga” region with a high-temperature climate and limited water availability, providing raw material for the manufacture of a wide variety of products in regions where agriculture is restricted [8,10]. Its pulp, which represents a negligible percentage of the fruit’s mass, is commonly ignored; however, the licuri nut stands out for its use [11].

The licuri nut is of great importance in the regions where it is located, as it represents a source of income for the population. Mainly, licuri nut is incorporated into the diet of beef cattle and animals that supply milk exported outside Brazil, as an alternative to feeding grains whose production is limited in the area [12,13]. However, it is important to note that exploitation still occurs only in an extractive way [14].

As a food ingredient, licuri is consumed in various ways, as an ingredient in regional preparations, in its natural, caramelized form, or even in a vegetable drink made from the fruit. Also, an oil used in cooking by the semi-arid population is extracted from the almond [14,15].

In the context of cereal bars, commercial formulations are commonly based on a mixture of cereals, mainly oats, combined with dried fruits or even oilseed fruits, as in the case of licuri [16]. Thus, the hypothesis created is that licuri, already consumed in different ways in the regions where it is cultivated, could be an interesting ingredient in cereal bars, from a commercial, sensorial, and nutritional point of view.

Commercially, the incorporation of licuri in a product with increasing consumption can exponentially increase its commercial value, adding to the populations that survive with its extraction; in addition, nutritionally being an almond rich in proteins (11.5 g/100 g) and with considerable energy value (240 kcal/100 g), it might be suitable for incorporation in cereal bars that aim to provide high nutritional density in a reduced portion [14,17].

Another point to be considered is the sustainable nature of licuri production, which requires little water and has reduced greenhouse gas (GHG) emissions, given that its production is exclusively extractive [18]. Furthermore, considering the sustainable development objectives stipulated by the United Nations Organization, the increased appreciation of licuri as a culinary ingredient is related to the fulfillment of several of these objectives, mainly those that refer to the consumption of local production and the valorization of products from family and organic farming [19].

However, although some studies already incorporated licuri nuts in different preparations such as bread and cake, no studies aimed to develop and describe the sensory profile of cereal bars incorporated with licuri nuts [14,15].

In this context, considering the demand for cereal bars and the need to incorporate licuri into preparations to make better use of this local production, this study aimed to produce cereal bars using different proportions of licuri nuts as a key ingredient, in addition to describing its sensory profile to determine the best proportion of incorporation of licuri nuts.

## 2. Materials and Methods

This study was conducted in four steps: (i) sample development, (ii) chemical composition analysis, (iii) sensory Analysis, and (iv) statistical analysis.

### 2.1. Sample Development

The samples of cereal bars were developed by the authors, in two stages, one consisting of the production of the clumping syrup and the other a mixture of dry ingredients. For the clumping syrup, the following ingredients were combined: invert sugar syrup (70%), sunflower oil (15%), glucose syrup (10%), and isomalt (5%).

Regarding the dry ingredients, different proportions of fined rolled oats (*Avena sativa)* and licuri nuts (*Syagrus coronata)* were combined to produce five different samples:

A control one, with only oats, four others with licuri nuts in the proportions of 25%, 50%, 75%, and the last one, with 100% of licuri oats. Then, the cereal bars were produced with 50% clumping syrup and 50% dry ingredients mixture. Table 1 below summarizes all sample compositions.

Then, the mixture of the clumping syrup with the different dry ingredients proportions was baked at 180 degrees, for twenty minutes. Cereal bars were cooled to room temperature, then portioned in bars with 15 g each and kept in hermetic recipients for better conservation. All samples of cereal bars were produced on the same day as the subsequent sensory analysis.

### 2.2. Chemical Composition Analysis

To analyze the chemical composition of the cereal bars developed, the management instrument created by Camargo et al. (2023), called technical preparation card, was used [20]. This instrument systematizes the ingredients used in preparations and based on nutritional composition tables, estimates the nutritional value of authorial recipes.

In this sense, the energy value of cereal bars (kcal) was estimated, as well as the values of carbohydrates (g), proteins (g), lipids (g), and dietary fiber (g). The nutritional value of licuri nuts was estimated based on values found in the literature [10].

### 2.3. Sensory Analysis

The sensory analysis was conducted to list applicable descriptive characteristics and evaluate the acceptance of developed cereal bars. Both descriptive and acceptance analyses were assessed at the same time, in the University Center IESB sensory evaluation lab, from 1 pm to 8 pm, on 29 November 2023.

The acceptance test was performed using a structured 9-point hedonic scale, where 1 is equivalent to extreme dislike, 5 to neither dislike or like, and 9 to extreme liking [21]. In this phase, the following attributes were assessed: appearance, texture, flavor, aroma, and overall acceptance. To evaluate its acceptance, the following cutoff points were adopted: average evaluations with values equal to or below 5 were considered as disliking; averages equal to 5 as indifferent and equal to or above 6 as acceptance [21].

The check-all-that-apply (CATA) test was performed to list descriptive characteristics of produced cereal bars. All sensory and non-sensory descriptors for the CATA test were elaborated based on Kelly’s Repertory Grid Method [22], with the presence of five assessors, all with 500 h minimum experience related to the development of food products.

In total, four sessions of 30 min each were conducted, and with aid of an expert panel moderator, 60 descriptors were raised by the method. Of these descriptors, 12 were raised for describing appearance, 15 for aroma, 5 for color, 15 for flavor, and 13 for texture.

Regarding the panelists, all participants were recruited randomly from the university center campus. Before the tests, the panelists were informed of the aims of the study and signed an informed consent form. The project was approved by the University Center IESB ethics committee (Register number: 75048323.1.0000.8927).

A recruitment questionnaire was handed to panelists to collect sociodemographic information, frequency of consumption of cereal bars, presence of food allergies, and willingness to participate. Samples were coded with random non-consecutive 3-digit codes.

In the given forms, both samples’ orders and CATA descriptors were presented in a randomized and balanced order to prevent sensory overload, resulting in bias in the evaluation. Further, 5 g of each sample was served in disposable white plates, while panelists also received two glasses, one with room temperature water and the other for disposal of samples if needed.

Subsequently, panelists completed the given forms according to their perception of hedonic attributes and applicable CATA descriptors. A total of 117 untrained panelists participated in the sensory analysis.

### 2.4. Statistical Analysis

For analysis of the acceptance test, an analysis of variance (ANOVA) with 95% significance level (*p* < 0.05) was performed to find possible differences between evaluated attributes among samples. Then, a post hoc Fisher’s HSD test, also with 95% significance level, was carried out. Also, radar graphics were generated to visualize the distribution of acceptance of each evaluated attribute for each sample.

The CATA analysis began by comparing descriptors using the non-parametric Cochran’s Q test (*p* < 0.001) to identify significant differences in consumer perceptions of various attributes across different samples. Subsequently, a multiple pairwise comparison test was conducted using the Bonferroni (McNemar) procedure with a significance level of 5% (*p* < 0.05).

Additionally, a correspondence analysis (CA) based on chi-squared distances was employed to create a sensory map of the samples. A test of independence between rows and columns was carried out at a 5% significance level (*p* < 0.05). Xlstat^®^ (Addinsoft, Paris, France, 2018) was used to perform all statistical analyses.

## 3. Results and Discussion

The chemical composition of 100 g of cereal bars is shown in Table 2, below.

In general, given the lipid-rich nature of licuri nuts, the lipid content of cereal bars increased proportionally as the percentage of licuri in the bars increased [10]. This also increased the energy value of the bars. However, it is important to highlight that despite the increase in energy value, an increase was also observed in terms of dietary fiber content, while the carbohydrate and protein values were reduced.

Considering the common nutritional composition of commercial cereal bars, carbohydrates stand out as predominant nutrients, mainly given the sugar content and the use of cereals, such as oats, rice flakes, or quinoa [17]. Commonly, cereal bars can contain between 50 and 75% of their weight in sugar, compared to the bar developed in this study, which used a minimum proportion of 50% [17]. In this way, one of the potential benefits of the licuri nut bar is to provide a product reduced in sugar and increased in dietary fiber and can be included in diets that restrict the aforementioned nutrients.

As another potential benefit, the content of antioxidant phenolic compounds in licuri nuts stands out, whose average value is around 75.48 g.g DPPH^−1^, also highlighting its stability at high temperatures [23].

At the end of the sensory analysis, a total of 122 panelists participated through random recruitment on the university campus. Of these, all reported habitual consumption of both coconut and cereal bars (At least 1× per week), and no panelists were lost due to the exclusion criteria used.

Of these, 45% were between 18 and 25 years old, 22% between 26 and 35 years old, 16% between 36 and 50 years old, and 15% were over 50 years old. Regarding gender distribution, most panelists were female, representing 59% of the sample, while male panelists comprised 41% of the total.

**Figure 1:**
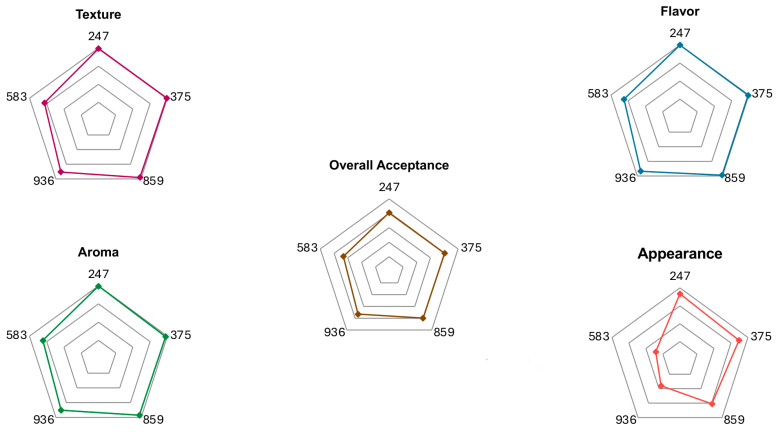
Radar graphs generated from evaluated attributes on the acceptance test for each sample.

The results of the acceptance analysis of cereal bars incorporated with licuri are shown in Table 3 and Figure 1 below.

From the sensory analysis, it was possible to observe that the introduction of licuri nuts was successful in all formulated samples, given that all attributes presented average evaluations above 6 [21,24]. However, higher overall acceptance levels with no significant differences between them were found in samples incorporated with 100% (247), 75% (375), and 50% (859) of licuri, highlighting licuri’s nut potential to be implemented as an ingredient in cereal bars. Furthermore, when observing the radar graphs generated from the acceptance data, it is possible to observe that the vertices referring to these same samples (247, 375, and 859) present visible symmetry, thus denoting the absence of significant differences [25]

It is worth noting that even regional products subject to sensory analysis tend to be well accepted, and those rich in sugar, such as cereal bars, usually present good purchasing intentions [26]

The licuri nut is phylogenetically close to the coconut (*Cocus nucifera* L.), thus presenting a characteristic flavor and aroma like conventional coconut [10,15]. Coconut, in turn, is already widely incorporated into cereal bars successfully, mainly given the sensory characteristics it provides in preparations [27]. As an oleaginous fruit, when incorporated into preparations, it provides well-accepted sensorial characteristics, such as lubricity, pleasant aftertaste, shine, and unctuousness [10].

However, a limitation in the use of coconut refers to its cultivation. Demanding environments with sandy soil and a high content of mineral salts and humidity, typical of coastal areas, mean that its mostly extractive cultivation is restricted to locations close to beaches [28]. In this sense, this ingredient is more economically viable in tropical regions, as evidenced in studies that used this fruit in cereal bars produced in regions of Africa, Brazil, and Asia [27].

The licuri, in turn, is adapted to both hotter and colder regions; however, originating from the Brazilian *Caatinga*, it is a specimen adapted to low relative humidity (<25%) and rigid soils, reddish due to the presence of ore of iron, presenting more viable cultivation in regions away from the coast [11]. Such soils stand out for being ideal for growing berries common to the northern hemisphere’s eating habits, such as blueberries and cranberries that require more iron [29]. In this sense, one of the potential uses of licuri is to replace traditional coconut in preparations such as granola and cereal bars.

Given the high number of descriptors (60), 12 of them were excluded given that there were no frequencies associated with these, resulting in a final 48 descriptors. The presence of significant differences among descriptors and their frequencies highlights the dependence between rows and columns. A contingency table containing the absolute frequencies of signed descriptors and its respective *p*-values from Cochran’s Q test is shown in Table 4.

A total of 41 descriptors (85.41%) presented significant differences among their respective frequencies, thus discriminating samples, and suggesting heterogeneity between the panelists’ perceptions of the different cereal bars produced. Among all evaluated modalities, discrepancies were found in 72.72% (n = 8) of descriptors for appearance, 88.88% (n = 8) for aroma, 100% (n = 5) for color, 91.66% (n = 11) for flavor and 81.81% (n = 9) for texture. From all descriptors, the following did not present significant differences between samples: “Irregular appearance”, “Fibrous appearance”, “Heterogeneous appearance”, “Sweet aroma’, “Rancid taste”, “Dense texture”, and “Granular texture”.

**Figure 2:**
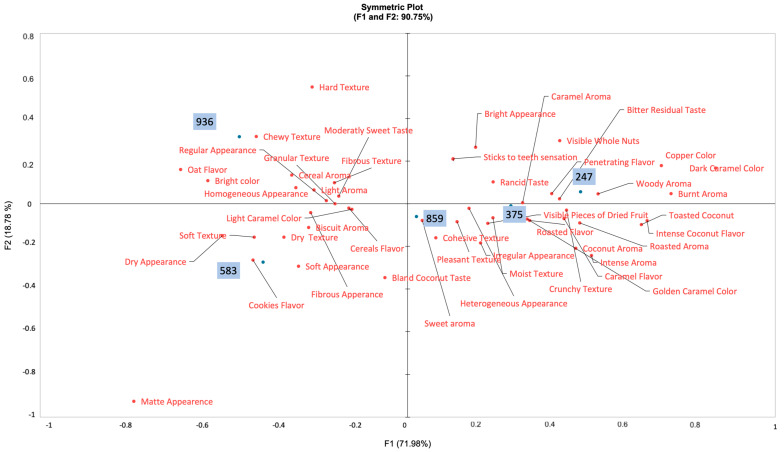
Preference map generated based on chi-squared distances between evaluated descriptors and overall acceptance of the developed cereal bars. In red are the descriptors, and samples are in blue. 247 (100% of licuri nuts); 375 (75% of licuri nuts); 859 (50% of licuri nuts); 936 (0% of licuri nuts); 583 (25% of licuri nuts).

A preference map generated based on chi-squared distances between evaluated descriptors and overall acceptance of the developed cereal bars is shown in Figure 2.

Within the context of the descriptors of all evaluated modalities, the different percentages of incorporation of licuri nuts were determining factors for the acceptance obtained in the bars developed, given that descriptors placed on the positive side of the F1 axis (which explains 71.98% of the variance) presented higher frequencies in samples with more incorporated licuri nut, such as samples 859 (50%), 375 (75%), and 859 (100%) (Table 3 and Figure 2).

For the appearance modality, descriptors, such as “Bright appearance”, “Visible whole nuts”, “Bright appearance”, “Heterogenous appearance”, and “Irregular appearance”, were present in the positive side of the F1 axis, more frequently associated with bars with concentrations of licuri nut starting from 50% up to 100%, in samples 859, 375, and 247, respectively. On the other hand, descriptors, such as “Regular appearance”, “Dry appearance”, and “Soft appearance”, are associated with bars with lower levels of incorporated licuri nuts, such as samples 583 and 936, with 25% of licuri nuts and 0%, also appearing on the negative side of the F1 axis.

The appearance of a product is a determining factor in its purchase intention, both in products aimed at children and those aimed at adults [16,30]. In the case of the developed bars, the presence of the licuri positively influenced the acceptance of the bars. For example, as it is a small (5 to 10 mm) and round nut, its incorporation into cereal bars was in its entire form, without cutting or crushing, which possibly justifies the frequency of descriptors related to heterogeneity and irregularity of the cereal bars, as well as the perception of the presence of nuts (as evidenced in “Visible whole nuts”) [31].

The “shininess” (evident in “Bright appearance”), in turn, results both from the incorporation of the licuri nut, which, being an oilseed, exudes part of its oily content during cooking, and also from the clumping syrup used, whose caramelization also contributes to the presence of this attribute [32].

In another study, also carried out with cereal bars, the dry appearance negatively influenced the acceptance of the developed bar, in a similar way to that found in our study, reinforcing that this attribute is undesirable in the case of cereal bars [33]. In the case of the cereal bars developed, the descriptors that negatively influenced the acceptance of the bars possibly derive from the greater proportion of oats, which become soft and integrate as a result of rheological phenomena inherent to their cooking, such as gelatinization and dextrinization [34,35].

For aroma, descriptors within the positive axis were also associated with samples 859, 375, and 247, as “Caramel aroma”, “Roasted aroma”, “Woody aroma”, “Burnt aroma”, “Sweet aroma”, “Coconut aroma“, and “Intense aroma”. In samples, 583 and 936, the most frequently associated descriptors were “Cereal aroma”, “Light aroma”, and “Biscuit aroma”. In the case of cereal bars, other descriptive studies have already demonstrated that the aroma is also decisive in their acceptance [16,33,36]. Commonly being baked and sweet preparations, the ingredients incorporated in these products contribute in different ways to the final aroma of the product.

Sugar, used as a binder for dry ingredients, has a characteristic aroma resulting from its caramelization, which occurs during the cooking process of cereal bars [37]. Furthermore, the incorporated ingredients, such as fruits and oilseeds, have volatile compounds with characteristic aromas, as in the case of licuri nuts [38,39].

In a study that used licuri nuts in bread, the aroma of samples that contained higher proportions of licuri nuts presented higher scores compared to samples with lower proportions of nuts [40]. Thus, the descriptors such as “Caramel aroma” and “Sweet aroma” are possibly related to the sugar incorporated in the clumping syrup, while the other descriptors positively associated with the acceptance of cereal bars are related to the cooking of licuri nuts and the release of its aromatic compounds, as evidenced in the descriptors “Coconut aroma”, “Roasted aroma”, and “Woody aroma” [39,41].

Regarding color, descriptors located on the positive F1 axis were “Copper color”, “Dark caramel color”, and “Golden caramel color”, being associated with the overall acceptance of samples 859, 375, and 247, as other evaluated descriptors. Samples located on the negative side of F1 axis (583 and 936) were most associated with color descriptors such as “Light caramel color” and “Bright color”. The licuri nut has shades that vary from light brown to dark brown, which tend to darken during the cooking process [11]. In this sense, it is likely that the licuri nut is responsible for the higher frequency of descriptors positively associated with the acceptance of bars with higher licuri content.

As for the flavor modality, samples 859, 275, and 247 were associated with “Bitter residual taste”, “Penetrating flavor”, “Rancid taste”, “Roasted flavor”, and “Intense coconut flavor”, noting that all these descriptors were located on the positive side of the F1 axis. However, similar to that noted with other modalities, descriptors located on the negative side of the F1 axis were associated with samples 583 and 936, such as “Oat flavor”, “Moderately sweet taste”, “Cookies Flavor”, and “Cereal flavor”. Although flavor is not the first sensory modality to be perceived when tasting a product, it is decisive in aspects related to purchase intention and customer loyalty to the evaluated product [42,43].

It is noteworthy that the development of products with regional foods is challenging, as the flavors of these foods are not common in widely consumed products [44,45]. In the context of the bars developed, the descriptor “Intense coconut flavor” was the most present, being a direct consequence of the incorporation of licuri nuts, which have a flavor similar to traditional coconut [16]. In contrast, descriptors negatively associated with bar acceptance are common in cereal-based products, such as oats.

However, one point to highlight is the presence of sugar in the cereal bars developed. Sugary products are the most accepted by the general population, and in the case of other studies related to the development of cereal bars, the presence of this ingredient was associated with positive descriptors [46,47].

Finally, regarding the texture modality, with the same tendency as aforementioned modalities, positive descriptors associated with samples 247, 375, and 936 were “Sticks to teeth sensation”, “Cohesive texture”, “Pleasant texture”, “Crunchy texture”, and “Moist texture”, whereas on the negative side, descriptors were “Chewy texture”, “Fibrous texture”, “Granular texture”, “Soft texture”, and “Dry texture”. Oilseeds in general have sensory characteristics, such as crunchiness, greasiness, and moisture; therefore, it is likely that the presence of licuri is responsible for the presence of descriptors positively associated with the acceptance of cereal bars incorporated with a higher proportion of licuri [48]

In general, with regard to the acceptance of the cereal bars developed, the presence of licuri nuts stood out as being determined based on their acceptance, since despite there being no significant differences in relation to the acceptance of all the bars developed, the samples with a higher proportion of licuri (247, 375, and 936) presented both higher scores and descriptors associated with the presence of licuri nuts located on the acceptance axis of the preference map.

Such results are promising, since despite being incorporated into regional preparations, the greatest economic value related to licuri is found in its incorporation into animal feed [12,49]. Furthermore, as it is a product derived from extractivism in economically vulnerable Brazilian populations, the wider use of licuri nuts is in line with sustainable development objectives [19].

Thus, despite not exhausting the subject in any way, the present study highlights a possibility of incorporating a regional Brazilian ingredient into a product of widespread consumption, acceptance, and commercialization such as cereal bars, thus expanding the use of licuri and its socioeconomic potential.

One of the study’s main limitations is the lack of characterization of the chemical composition of the cereal bars developed. Food composition tables are validated methods for describing the chemical composition of foods, also accepted in Brazilian law [37].

However, although the literature already highlights the multiple benefits of incorporating this ingredient, laboratory analyses would provide greater robustness to the nutritional viability of this product [11,48]. Based on the acceptance of bars with different proportions of licuri, it is hoped to stimulate this production and subsequent analysis. Being an important cultural product in several communities in regions of Brazil, the addition of another preparation can result in greater socioeconomic value [31].

## 4. Conclusions

Regarding the chemical composition, cereal bars incorporated with licuri nuts presented higher energy and lipid concentration as the proportion of implemented licuri nuts increased. However, the dietary fiber content increased with the incorporation of licuri nuts.

This study demonstrated that cereal bars incorporated with licuri nuts were well accepted, independent of the proportion of licuri nuts incorporated. However, the rapid descriptive analysis demonstrated that descriptors associated with the presence of licuri nuts were positively associated with product acceptance. In contrast, traditional characteristics of bars with a lower percentage of licuri or just oats presented descriptors less associated with product acceptance.

Given the socioeconomic importance of licuri in the regions of Brazil where it is grown, this study demonstrated that this ingredient could be successfully incorporated into cereal bars, one of the preparations whose consumption is growing worldwide.

## Figures and Tables

**Table 1:** Proportion of ingredients implemented in developed samples of cereal bars.

Sample	Code	Clumping Syrup	Rolled Oats	Licuri Nuts	% of Substitution
1 (Control)	936	50%	50%	0%	0%
2	583		37.5%	12.5%	25%
3	859		25%	25%	50%
4	375		12.15%	37.5%	75%
5	247		0%	50%	100%

**Table 2:** Chemical composition of 100 g of developed cereal bars.

Sample	Energy Value (Kcal)	Carbohydrates (g/100 g)	Protein (g/100 g)	Lipids (g/100 g)	Dietary Fiber (g/100 g)
936 (0% of Licuri Nuts)	413 kcal	79.40 g	8.00 g	6.88 g	4.90 g
583 (25% of Licuri Nuts)	430 kcal	72.75 g	7.44 g	12.24 g	5.55 g
859 (50% of Licuri Nuts)	448 kcal	65.10 g	6.88 g	17.60 g	7.31 g
375 (75% of Licuri Nuts)	465 kcal	58.24 g	6.32 g	22.96 g	8.12 g
247 (25% of Licuri Nuts)	482 kcal	51.04 g	5.75 g	28.31 g	9.42 g

**Table 3:** Means and standard deviations of the evaluated attributes on the acceptance test.

Sample	Appearance	Flavor	Aroma	Texture	Overall Acceptance
247 (100% of Licuri Nuts)	8.34 ± 1.06 ^a^	7.99 ± 1.40 ^a^	7.97 ± 1.27 ^a^	7.93 ± 1.50 ^a^	8.11 ± 1.16 ^a^
375 (75% of Licuri Nuts)	8.24 ± 0.95 ^a^	7.91 ± 1.35 ^a^	7.80 ± 1.25 ^a^	7.93 ± 1.19 ^a^	8.05 ± 1.05 ^a^
859 (50% of Licuri Nuts)	8.02 ± 1.33 ^a^	7.89 ± 1.35 ^a^	7.70 ± 1.39 ^a^	7.81 ± 1.27 ^a^	7.98 ± 1.16 ^a^
936 (0% of Licuri Nuts)	7.40 ± 1.81 ^b^	7.36 ± 1.8 ^b^	7.02 ± 2.01 ^b^	7.07 ± 2.16 ^b^	7.30 ± 1.87 ^b^
583 (25% of Licuri Nuts)	7.21 ± 1.60 ^b^	6.49 ± 1.60 ^c^	6.45 ± 1.59 ^c^	6.27 ± 1.69 ^c^	6.65 ± 1.57 ^c^

Means with different superscript letters in the same column statistically differ at a 95% confidence level (*p* < 0.05, Tukey’s HSD test).

**Table 4:** List of sensory descriptors utilized in CATA evaluation, with *p*-values from Cochran’s Q test.

Descriptors/Samples	247	375	583	859	936	*p* Value
Appearance						
Regular Appearance	32 ^a^	44 ^ab^	56 ^b^	46 ^ab^	55 ^b^	**0.001**
Visible Whole Nuts	48 ^d^	35 ^c^	5 ^a^	21 ^b^	24 ^b^	**<0.0001**
Irregular Appearance	16 ^a^	15 ^a^	13 ^a^	17 ^a^	5 ^a^	0.050
Soft Appearance	7 ^ab^	8 ^ab^	20 ^b^	13 ^ab^	9 ^ab^	**0.006**
Visible Pieces of Dried Fruit	43 ^b^	41 ^b^	30 ^ab^	33 ^ab^	17 ^a^	**<0.0001**
Bright Appearance	102 ^c^	98 ^bc^	28 ^a^	83 ^b^	97 ^bc^	**<0.0001**
Homogeneous Appearance	16 ^a^	18 ^a^	29 ^ab^	25 ^ab^	33 ^b^	**0.005**
Fibrous Appearance	20 ^a^	19 ^a^	36 ^a^	26 ^a^	30 ^a^	0.021
Matte Appearance	3 ^a^	2 ^a^	56 ^c^	16 ^b^	6 ^ab^	**<0.0001**
Heterogeneous Appearance	26 ^a^	19 ^a^	16 ^a^	23 ^a^	13 ^a^	0.084
Dry Appearance	8 ^a^	12 ^ab^	34 ^c^	13 ^ab^	23 ^bc^	**<0.0001**
Aroma						
Coconut Aroma	78 ^c^	73 ^c^	40 ^b^	56 ^bc^	3 ^a^	**<0.0001**
Biscuit Aroma	15 ^ab^	11 ^a^	28 ^b^	23 ^ab^	20 ^ab^	**0.011**
Sweet aroma	51 ^a^	46 ^a^	49 ^a^	52 ^a^	34 ^a^	0.057
Caramel Aroma	23 ^ab^	28 ^b^	11 ^a^	18 ^ab^	10 ^a^	**0.001**
Intense Aroma	17 ^b^	12 ^b^	9 ^ab^	6 ^ab^	0 ^a^	**0.000**
Light Aroma	33 ^a^	34 ^a^	52 ^ab^	48 ^ab^	58 ^b^	**0.000**
Roasted Aroma	21 ^b^	19 ^b^	8 ^ab^	14 ^ab^	13 ^a^	**<0.0001**
Cereal Aroma	32 ^a^	41 ^ab^	57 ^bc^	44 ^ab^	73 ^c^	**<0.0001**
Woody Aroma	15 ^b^	9 ^ab^	4 ^ab^	5 ^ab^	3 ^a^	**0.005**
**Color**						
Light Caramel Color	20 ^a^	34 ^ab^	35 ^ab^	46 ^b^	34 ^ab^	**0.005**
Copper Color	15 ^b^	12 ^ab^	1 ^a^	4 ^ab^	3 ^ab^	**0.000**
Golden Caramel Color	28 ^b^	32 ^b^	15 ^ab^	24 ^ab^	9 ^a^	**0.000**
Dark Caramel Color	44 ^d^	23 ^c^	1 ^a^	11 ^bc^	4 ^ab^	**<0.0001**
Bright color	16 ^a^	32 ^ab^	72 ^c^	48 ^b^	86 ^c^	**<0.0001**
Flavor						
Oat Flavor	16 ^a^	27 ^a^	80 ^c^	53 ^b^	104 ^d^	**<0.0001**
Penetrating Flavor	25 ^b^	19 ^ab^	8 ^a^	15 ^ab^	8 ^a^	**0.001**
Cereal Flavor	40 ^a^	57 ^ab^	65 ^b^	65 ^b^	60 ^ab^	**0.002**
Caramel Flavor	29 ^bc^	29 ^c^	12 ^ab^	20 ^abc^	6 ^a^	**<0.0001**
Bitter Residual Taste	22 ^b^	8 ^ab^	7 ^a^	10 ^ab^	5 ^a^	**0.000**
Rancid Taste	18 ^a^	8 ^a^	8 ^a^	6 ^a^	8 ^a^	0.019
Roasted Flavor	22 ^b^	19 ^ab^	11 ^ab^	17 ^ab^	6 ^a^	**0.007**
Intense Coconut Flavor	52 ^d^	45 ^cd^	11 ^b^	31 ^c^	1 ^a^	**<0.0001**
Toasted Coconut Flavor	64 ^d^	34 ^c^	16 ^b^	31 ^bc^	1 ^a^	**<0.0001**
Bland Coconut Taste	16 ^ab^	36 ^c^	41 ^c^	35 ^bc^	12 ^a^	**<0.0001**
Moderately Sweet Taste	33 ^a^	42 ^ab^	50 ^ab^	45 ^ab^	52 ^b^	**0.037**
Cookies Flavor	4 ^a^	10 ^ab^	21 ^b^	9 ^ab^	11 ^ab^	**0.002**
Texture						
Hard Texture	11 ^ab^	4 ^a^	5 ^a^	9 ^ab^	20 ^b^	**0.001**
Pleasant Texture	71 ^bc^	75 ^bc^	58 ^ab^	79 ^c^	39 ^a^	**<0.0001**
Soft Texture	10 ^a^	15 ^a^	34 ^b^	13 ^a^	22 ^ab^	**<0.0001**
Fibrous Texture	38 ^ab^	29 ^a^	45 ^ab^	30 ^a^	50 ^b^	**0.001**
Cohesive Texture	14 ^ab^	25 ^b^	18 ^ab^	25 ^b^	10 ^a^	**0.004**
Sticks to teeth sensation	45 ^b^	26 ^a^	20 ^a^	21 ^a^	32 ^ab^	**0.000**
Dry Texture	12 ^a^	13 ^a^	31 ^b^	19 ^ab^	20 ^ab^	**0.001**
Dense Texture	15 ^a^	19 ^a^	7 ^a^	13 ^a^	15 ^a^	0.071
Crunchy Texture	47 ^b^	46 ^b^	15 ^a^	41 ^b^	11 ^a^	**<0.0001**
Chewy Texture	11 ^a^	18 ^a^	21 ^ab^	15 ^a^	39 ^b^	**<0.0001**
Granular Texture	24 ^a^	15 ^a^	31 ^a^	26 ^a^	28 ^a^	0.044

Different superscript letters in the same row present significant statistical differences (*p* < 0.05); *p* values highlighted in bold represents *p* values associated with significant differences. 247 (100% of licuri nuts); 375 (75% of licuri nuts); 859 (50% of licuri nuts); 936 (0% of licuri nuts); 583 (25% of licuri nuts).

## Data Availability

Data are contained within the article.

## References

[1] Kaur S., Das M. (2011). Functional Foods: An Overview. Food Sci. Biotechnol..

[2] Orrego C.E., Salgado N., Botero C.A. (2014). Developments and Trends in Fruit Bar Production and Characterization. Crit. Rev. Food Sci. Nutr..

[3] Rosa Machado A.M., Galdeano M.C., Freitas de Sá D.d.G.C., Fraga de Souza E., de Alcantara M., Cordeiro de Freitas S., Tonon R.V. (2023). Red Wine Processing-Derived Brazilian Alicante Bouschet Grape Skin as a Promising Ingredient for Cereal Bars Production. Food Sci. Technol. Int..

[4] Carvalho V.S., Conti-Silva A.C. (2018). Cereal Bars Produced with Banana Peel Flour: Evaluation of Acceptability and Sensory Profile. J. Sci. Food Agric..

[5] de Carvalho M.G. (2011). Formulation and Sensory Acceptance of Cereal-Bars Made with Almonds of Chichá, Sapucaia and Gurguéia Nuts. Open Food Sci. J..

[6] (2023). Mordor Intelligence Cereal Bar Market—Share & Industry Analysis.

[7] da Silva É.C., Sobrinho V.d.S., Cereda M.P. (2013). Stability of Cassava Flour-Based Food Bars. Food Sci. Technol..

[8] de Souza F.G., de Araújo F.F., de Paulo Farias D., Zanotto A.W., Neri-Numa I.A., Pastore G.M. (2020). Brazilian Fruits of Arecaceae Family: An Overview of Some Representatives with Promising Food, Therapeutic and Industrial Applications. Food Res. Int..

[9] de Carvalho A.P.A., Conte-Junior C.A. (2021). Health Benefits of Phytochemicals from Brazilian Native Foods and Plants: Antioxidant, Antimicrobial, Anti-Cancer, and Risk Factors of Metabolic/Endocrine Disorders Control. Trends Food Sci. Technol..

[10] Crepaldi I.C., Bicudo L., Almeida-Muradian D.E., Dias M., Rios G., De Vuono M., Penteado C., Salatino A.A. (2001). Composição Nutricional Do Fruto de Licuri (Syagrus Coronata (Martius) Beccari). Braz. J. Bot..

[11] De Andrade W.M., Alves Ramos M., Silva Souto W.M., Bento-Silva J.S., De Albuquerque U.P., De Lima Araújo E. (2015). Knowledge, Uses and Practices of the Licuri Palm (Syagrus Coronata (Mart.) Becc.) around Protected Areas in Northeastern Brazil Holding the Endangered Species Lear’s Macaw (Anodorhynchus Leari). Trop. Conserv. Sci..

[12] Lima L.d.S., Oliveira R.L., Bagaldo A.R., Neto A.F.G., Barbosa L.P., Borja M.S. (2011). Production Performance of Lactating Dairy Cows at Pasture Fed Concentrate Supplemented with Licuri Oil. Revista Brasileira de Zootecnia.

[13] De Gouvêa A.L.L. (2014). Qualidade da Carne e dos Produtos Cárneos de Tourinhos Anelorados submetidos a Dietas com Torta de Licuri. Ph.D. Thesis.

[14] Antoniassi R., de Freitas S.C., de Oliveira S.P., Vieira T.M.F.S., Bizzo H.R., Matsuura M.I.d.S.F., Miranda P.C. Valor Nutricional Da Amêndoa de Licuri (Syagrus Coronata) Utilizada Em Preparações Culinárias Na Região Do Semi-Árido Baiano. Proceedings of the 6th Simpósio Latino Americano de Ciência de Alimentos.

[15] Castro D., Rybka A.C. (2018). Aceitação Sensorial de Doce de Umbu Com Amêndoas de Licuri. Boletim Embrapa Semiáridos.

[16] Bchir B., Jean-François T., Rabetafika H.N., Blecker C. (2018). Effect of Pear Apple and Date Fibres Incorporation on the Physico-Chemical, Sensory, Nutritional Characteristics and the Acceptability of Cereal Bars. Food Sci. Technol. Int..

[17] Eke-Ejifor J., Okoye C. (2019). Nutrient Composition, Lipid Profile and Sensory Properties of Cereal Bar Made from Locally Available Cereals and Nuts. Int. J. Biotechnol. Food Sci..

[18] Brasil, Ministério da Agricultura, Boas Práticas de Manejo Para o Extrativismo Sustentável Orgânico. https://www.gov.br/agricultura/pt-br/assuntos/sustentabilidade/organicos/arquivos-publicacoes-organicos/boas_praticas_de_manejo_para_o_extrativismo_sustentavel_organico_do_licuri.pdf.

[19] Roma J.C. (2019). Os Objetivos de Desenvolvimento Do Milênio e Sua Transição Para Os Objetivos de Desenvolvimento Sustentável. Cienc. Cult..

[20] Camargo E., Botelho R.B.A., Zandonadi R.P., Camargo E. (2023). Técnica Dietética, Pré-Preparo e Preparo de Alimentos—Manual De Laborátorio.

[21] Lim J. (2011). Hedonic Scaling: A Review of Methods and Theory. Food Qual. Prefer..

[22] Moskowitz H.R. (1983). Product Testing and Sensory Evaluation of Foods: Marketing and R&D Approaches.

[23] Elizabeth K., Souza D.E., Qualidade M., Atividade E., De Fruto A. (2011). Qualidade e Atividade Antioxidante de Fruto e Seu Óleo de Genótipos Do Licurizeiro (Syagrus Coronata). Ph.D Thesis.

[24] Bergara-Almeida S., Aparecida M., Da Silva A.P. (2002). Hedonic Scale with Reference: Performance in Obtaining Predictive Models. Food Qual. Prefer..

[25] Saary M.J. (2008). Radar Plots: A Useful Way for Presenting Multivariate Health Care Data. J. Clin. Epidemiol..

[26] Macedo I.B., Romão de Lima B., Botelho R., Alencar E.R., Zandonadi R. (2019). Dried Apples as Substitute for Refined Sugar in Pound Cakes. J. Acad. Nutr. Diet..

[27] Joy E.-E., Ellen Aswei B., Mbarabari Nicholas G. (2016). Preparation and Evaluation of Granola-a Breakfast Cereal, Sustituted with Maize (Zea May) and Coconut (Cocos Nucifera) Blend. Int. J. Nutr. Food Sci..

[28] Kalaiyarasi H.M., Raj K.S. (2022). Coconut Tree (Cocos Nucifera) Products: A Review of Global Cultivation and Its Benefits. Rev. Artic. J. Sustain. Environ. Manag..

[29] Tagliavini M., Abadía J., Rombolà A.D., Abadía A., Tsipouridis C., Marangoni B. (2000). Agronomic Means for the Control of Iron Deficiency Chlorosis in Deciduous Fruit Trees. J. Plant Nutr..

[30] Ueda J., Spence C., Okajima K. (2020). Effects of Varying the Standard Deviation of the Luminance on the Appearance of Food, Flavour Expectations, and Taste/Flavour Perception. Sci. Rep..

[31] Dos J., Guimarães S., Shiosaki R.K., Louise M., Mendes M. (2021). Licuri (Syagrus Coronata): Characteristics, Importance, Potential and Perspectives of the Small Coconut from Brazil. Desenvolvimento e Meio Ambiente.

[32] Murakoshi T., Masuda T., Utsumi K., Tsubota K., Wada Y. (2013). Glossiness and Perishable Food Quality: Visual Freshness Judgment of Fish Eyes Based on Luminance Distribution. PLoS ONE.

[33] Ribeiro J.C., Santos C., Lima R.C., Pintado M.E., Cunha L.M. (2022). Impact of Defatting and Drying Methods on the Overall Liking and Sensory Profile of a Cereal Bar Incorporating Edible Insect Species. Future Foods.

[34] Hüttner E.K., Arendt E.K. (2010). Recent Advances in Gluten-Free Baking and the Current Status of Oats. Trends Food Sci. Technol..

[35] Aydin E., Gocmen D. (2011). Cooking Quality and Sensorial Properties of Noodle Supplemented with Oat Flour. Food Sci. Biotechnol..

[36] Bower J.A., Whitten R. (2000). Sensory Characteristics and Consumer Liking for Cereal Bar Snack Foods. J. Sens. Stud..

[37] Ministério da Saúde, Agência Nacional de Vigilância Sanitária (2020). Da Diretoria Colegiada—RDC 429 de 8 de Outubro de 2020.

[38] Belviso S., Ghirardello D., Giordano M., Sousa Ribeiro G., de Souza Alves J., Parodi S., Risso S., Zeppa G. (2013). Phenolic Composition, Antioxidant Capacity and Volatile Compounds of Licuri (Syagrus Coronata (Martius) Beccari) Fruits as Affected by the Traditional Roasting Process. Food Res. Int..

[39] Oliveira de Souza L.I., Bezzera-Silva P.C., do Amaral Ferraz Navarro D.M., da Silva A.G., dos Santos Correia M.T., da Silva M.V., de Figueiredo R.C.B.Q. (2017). The Chemical Composition and Trypanocidal Activity of Volatile Oils from Brazilian Caatinga Plants. Biomed. Pharmacother..

[40] Gomes M.D.J., Aplevicz K.S. (2021). Development and Sensory Analysis of Breads Made with Licuri Flour (Syagrus Coronata (Martius) Beccari). J. Culin. Sci. Technol..

[41] Milner L., Kerry J.P., O’Sullivan M.G., Gallagher E. (2020). Physical, Textural and Sensory Characteristics of Reduced Sucrose Cakes, Incorporated with Clean-Label Sugar-Replacing Alternative Ingredients. Innov. Food Sci. Emerg. Technol..

[42] Bayarri S., Martí M., Carbonell I., Costell E. (2012). Identifying Drivers of Liking for Commercial Spreadable Cheeses with Different Fat Content. J. Sens. Stud..

[43] Nadathur S.R., Carolan M. (2017). Flavors, Taste Preferences, and the Consumer: Taste Modulation and Influencing Change in Dietary Patterns for a Sustainable Earth. Sustainable Protein Sources.

[44] Li X.E., Jervis S.M., Drake M.A. (2015). Examining Extrinsic Factors That Influence Product Acceptance: A Review. J. Food Sci..

[45] Okajima K., Spence C. (2011). Effects of Visual Food Texture on Taste Perception. Iperception.

[46] Borges M.S., Biz A.P., Bertolo A.P., Bagatini L., Rigo E., Cavalheiro D. (2021). Enriched Cereal Bars with Wine Fermentation Biomass. J. Sci. Food Agric..

[47] Lara N.d.S., de Sousa M.M.M., Paola de Pádua Gandra F., de Angelis-Pereira M.C., Carneiro J.d.D.S., Pereira R.G.F.A. (2019). Development of a Functional Food Bar Containing Coffee. Br. Food J..

[48] Montebello N.d.P., Araújo W.M.C., Botelho R.B.A. (2018). Alquimia Dos Alimentos—Série Alimentos e Bebidas.

[49] Araújo J.F., Azevêdo L.C.d., Santana C.R.d.S., Campos L.D.F., Moreira J.A., Almeida M.B.d. (2022). Licuri Milk Production and Conservation Treatments. Int. J. Adv. Eng. Res. Sci..

